# Comparison Between PET-CT–Guided Neck Dissection and Elective Neck Dissection in cT1-2N0 Tongue Squamous Cell Carcinoma

**DOI:** 10.3389/fonc.2020.00720

**Published:** 2020-06-10

**Authors:** Fengjie Zhu, Shuhan Sun, Kai Ba

**Affiliations:** ^1^Department of Oral Medicine, The First Affiliated Hospital of Zhengzhou University, Zhengzhou, China; ^2^Department of Oral Medicine, The Third Affiliated Hospital of Zhengzhou University, Zhengzhou, China

**Keywords:** PET-CT, elective neck dissection, survival analysis, early stage tongue cancer, tongue squamous cell carcinoma

## Abstract

**Objective:** Neck management in cT1-2N0 tongue squamous cell carcinoma (SCC) remains controversial. Our goal was to compare the survival difference between PET-CT–guided neck dissection and elective neck dissection (END) for the treatment of cT1-2 tongue SCC.

**Methods:** Patients with surgically treated cT1-2N0 tongue SCC were retrospectively enrolled. These patients were divided into two groups. Group A: The decision of whether to perform neck dissection was mainly based on the results of preoperative PET-CT examinations. Group B: Patients received END treatment without preoperative PET-CT examinations. The study endpoints were regional control (RC) and disease-specific survival (DSS). The Kaplan–Meier method was used to calculate the survival rates.

**Results:** Group A consisted of 66 patients, and 16 patients underwent neck dissection owing to positive PET-CT results. Group B consisted of 169 patients. The 5-year RC rates in group A and group B were 86 and 87%, respectively, and the difference was not significant (*p* = 0.731). The 5-year DSS rates in group A and group B were 93 and 90%, respectively, and the difference was not significant (*p* = 0.583).

**Conclusions:** Neck dissection can be safely avoided when the PET-CT scan reveals no neck lymph node involvement.

## Introduction

Tongue squamous cell carcinoma (SCC) is the most common malignancy in the oral cavity, and surgery is usually the first choice of treatment. For cT1-2N0 tongue SCC, its occult metastasis rate varies with different countries and races (1–3). Usually, neck dissection is required if the metastasis rate is greater than 20%. Although current high-quality research has described that routine neck dissection improves the prognosis by distinguishing patients who need adjuvant radiotherapy (1, 4, 5), researchers argue that most patients with cT1-2N0 disease do not have pathologic lymph node metastasis, and they are over-treated and exposed to possible neck dissection–related complications (2, 3); more importantly, the survival benefit associated with routine neck dissection could be contributed to by the late treatment of the metastatic necks at initial treatment. Owing to the development of individualized treatment concepts, it is very important for us to identify patients with pathologic lymph node metastasis preoperatively to achieve both oncologic and functional outcomes. A number of scholars support the predictive value of the depth of invasion (DOI), perineural invasion (PNI), and lymphovascular invasion (LVI) (6–8). However, these data are not always able to be obtained preoperatively.

PET-CT has been widely used for detecting primary cancer disease and possible metastasis sites. Zhang et al. (9) describe that the overall sensitivity and specificity of PET-CT for predicting lymph node metastasis in cT1-2 oral SCC was 21.4% and 98.4%, respectively, with a negative predictive value of 99.1%. Myers et al. (10) presented an estimated overall sensitivity of PET-CT for the N0 neck of 78% with a specificity of 100%. Similar results were also reported by Zheng et al. (11), Lee et al. (12), and Wong et al. (13). All these findings suggest that there might be high reliability for PET-CT in assessing neck status in cT1-2 tongue SCC. Therefore, our goal was to compare the survival difference between PET-CT–guided neck dissection and elective neck dissection (END) for the treatment of cT1-2 tongue SCC.

## Patients and Methods

### Ethnic Consideration

The Zhengzhou University Institutional Research Committee approved our study, and all patients signed informed consent agreements for medical research before the initial treatment. All methods were performed in accordance with the relevant guidelines and regulations.

### Patient Selection

From January 2010 to December 2016, patients with surgically treated primary cT1-2N0 tongue SCC were retrospectively enrolled. Patients without follow-up information were excluded from the analysis. Data regarding demographic and pathologic information as well as follow-up data were extracted. There was no consensus on the standard treatment of a cN0 neck in cT1-2 tongue SCC in China; therefore, in our cancer center, PET-CT was selectively suggested for patients with early stage oral SCC considering the patient's demand and the surgeon's preference from 2008, and our medical team had verified the feasibility of PET-CT in treating early stage tongue SCC (14). Therefore, enrolled patients were divided into two groups. Group A: The decision of whether to perform neck dissection was mainly based on the results of preoperative PET-CT examinations; if a positive result was reported by PET-CT, a neck dissection was performed, and if not, a wait-and-see policy was conducted. Group B: Patients received END treatment without preoperative PET-CT examinations.

### Important Variable Definition

Smokers/drinkers were defined as patients who smoked/drank at diagnosis or who had stopped for <1 year prior to diagnosis (15, 16). All pathological sections were re-reviewed by at least two pathologists. PNI was considered to be present if tumor cells were identified within the perineural space and/or nerve bundle; LVI was positive if tumor cells were noted within the lymphovascular channels (17). The pathologic DOI was measured from the level of the adjacent normal mucosa to the deepest point of tumor infiltration regardless of the presence or absence of ulceration (18). The cT1-2 status was defined according to the AJCC 2018 classification, and the cN0 status was defined as no doubt of metastatic lymph nodes after palpation and ultrasound, CT, and MRI examinations.

### Treatment Proposal

All patients underwent complete tumor resection with a margin of at least 1 cm, and the sublingual gland as well as the adipose tissue in the floor of the mouth was simultaneously resected. The neck dissection field included levels 1–3. Patients were followed every 3 months within 2 years after the operation and every 6 months for 2 years after the operation until the fifth year after the operation. Immediate interference was performed if disease recurrence was suspected. Adjuvant radiotherapy was suggested if there was pathologic lymph node metastasis, positive margins, PNI, or LVI.

### Pathologic Analysis

Surgical specimens were immediately fixed in 10% formalin and then cut into pieces with a thickness of 5 mm and a side length of 1 cm by embedding in paraffin. Sections of a thickness of 5 um were obtained at 0.5-mm intervals and stained with haematoxylin and eosin. All pathologic sections had been reviewed by at least two pathologists. Immunohistochemistry was performed if there was difficulty in identifying malignant disease.

### PET-CT Examination

The PET-CT scans (GE Healthcare, Milwaukee, WI) were performed by several technicians. Patients had an empty stomach for at least 6 hours before examination with glucose levels <200 mg/dL, and 10–20 mCi of [^18^F] FDG dosed according to the weight was given to each patient. Diagnostic CT images and axial PET were obtained from the calvarial vertex through the upper thighs. Emission images were obtained after radiopharmaceutical injection 60 min later. During the CT scan, no contrast medium was used. The images were reconstructed with a 2.5-mm thickness slice. For every suspicious lesion, the isocontour region of interest centered on the maximum value pixel was drawn automatically with workstation tools generating the SUV max of the region. An SUV max cutoff of 2.5 MBq/g was used for FDG-avid primary tumors and lymph nodes on PET-CT.

### Statistic Analysis

Student's *t*-tests and chi-square tests were used to compare the clinical and pathologic variables between the two groups, and the Kaplan–Meier method was used to calculate the regional control (RC) rate and disease-specific survival (DSS) rate. The survival time was calculated from the date of surgery to the last follow-up, the date of the first regional recurrence, or the date of cancer-caused death. All statistical analyses were performed using SPSS 20.0, and *p* < 0.05 was considered to be significant.

## Results

### Demography and Pathologic Data

A total of 235 patients (172 males and 63 females) were included in the analysis, and the mean age was 54.8 (range: 31–79) years. There were 66 patients in group A and 169 patients in group B.

In group A, there were 52 (78.8%) males and 14 (21.2%) females, and the mean age was 55.7 years. A total of 42 (63.6%) and 29 (43.9%) patients were smokers and drinkers, respectively. cT1 and cT2 tumors were present in 25 (37.9%) and 41 (62.1%) patients, respectively. PNI and LVI were reported in eight (12.1%) and seven (10.6%) patients, respectively. The mean pathologic DOI was 4.8 mm. pT1 and pT2 tumors were present in 20 (30.3%) and 46 (69.7%) patients, respectively. Tumor differentiation was well distributed in 22 (33.3%) patients, moderately distributed in 35 (53.0%) patients, and poorly distributed in nine (13.6%) patients. Negative margins were achieved in all patients. Fourteen (21.2%) patients received postoperative radiotherapy (Table 1).

**Table 1:** Demographic and pathologic information in the two groups.

**Variables**	**Group A (*n* = 66)**	**Group B (*n* = 169)**	***p***
Age	55.7	54.4	0.884
Sex			
Male	52 (78.8%)	120 (71.0%)	
Female	14 (21.2%)	49 (29.0%)	0.170
Smoker	42 (63.6%)	111 (65.7%)	0.768
Drinker	29 (43.9%)	58 (34.3%)	0.170
Clinical tumor stage			
cT1	25 (37.9%)	45 (26.6%)	
cT2	41 (62.1%)	124 (73.4%)	0.090
Perineural invasion	8 (12.1%)	22 (13.0%)	0.853
Lymphovascular invasion	7 (10.6%)	17 (10.1%)	0.901
Pathologic tumor stage			
pT1	20 (30.3%)	40 (23.7%)	
pT2	46 (69.7%)	129 (76.3%)	0.295
Tumor differentiation			
Well	22 (33.3%)	53 (31.4%)	
Moderate	35 (53.0%)	99 (58.6%)	
Poor	9 (13.6%)	17 (10.1%)	0.649
Postoperative radiotherapy	14 (21.2%)	30 (17.8%)	0.541

In group B, there were 120 (71.0%) males and 49 (29.0%) females, and the mean age was 54.4 years. A total of 111 (65.6%) and 58 (34.3%) patients were smokers and drinkers, respectively. cT1 and cT2 tumors were present in 45 (26.6%) and 124 (73.4%) patients, respectively. PNI and LVI were reported in 22 (13.0%) and 17 (10.1%) patients, respectively. The mean pathologic DOI was 4.7 mm. pT1 and pT2 tumors were present in 40 (23.7%) and 129 (76.3%) patients, respectively. Pathologic lymph node metastasis occurred in 30 (17.8%) patients, and in these patients, N1 was present in 25 cases, and N2a was present in five cases. Tumor differentiation was well distributed in 53 (31.4%) patients, moderately distributed in 99 (58.6%) patients, and poorly distributed in 17 (10.1%) patients. Negative margins were achieved in all patients. Thirty (17.8%) patients received postoperative radiotherapy (Table 1).

The two groups had similar distributions regarding age, sex, smoking status, drinking status, clinical, and pathologic tumor stage, PNI, LVI, tumor differentiation, and postoperative radiotherapy (Table 1, all *p* > 0.05).

### PET-CT–Guided Neck Dissection

In group A, 16 (9.5%) patients underwent neck dissection owing to positive PET-CT results, and postoperative pathology confirmed occult metastasis in 14 patients. Of the 14 patients, N1 was present in 12 patients, and N2a was present in two patients.

### Survival Analysis

**Figure 1:**
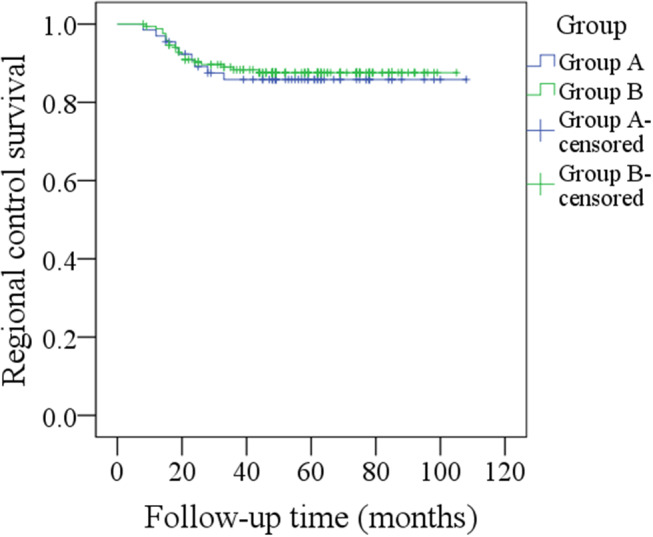
Comparison of regional control between the two groups (*p* = 0.731).

**Figure 2:**
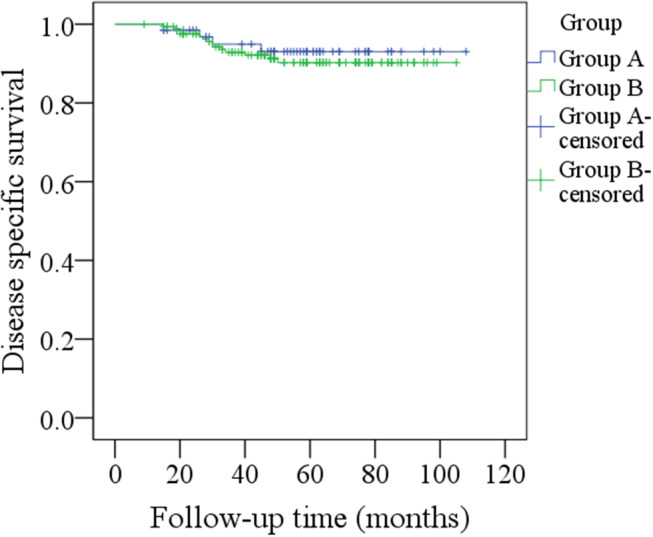
Comparison of disease-specific survival between the two groups (*p* = 0.583).

During our follow-up with a mean time of 48.5 months, in group A of the 66 patients, regional recurrence occurred in nine patients: level I occurrence in four patients, level II in three patients, and level III in two patients (Table 2). Among these nine patients, seven had a pathologic N+ neck at the initial treatment, two patients had not previously received neck dissection owing to previous negative PET-CT results, there was no local recurrence or distant metastasis, the 5-year RC rate was 86%, recurrent disease was successfully salvaged in six patients with radical operations, four patients died of the disease, and the 5-year DSS rate was 93%. In group B, local recurrence occurred in five patients, and regional recurrence occurred in 20 patients: level I recurrence in nine patients, level II in six patients, level IV in three patients, and level V in two patients (Table 2). Among these 20 patients, 14 had a pathologic N+ neck at the initial treatment, there was no distant metastasis, the 5-year RC rate was 87%, patients with local recurrence were all successfully salvaged with radial operations, regional recurrent disease was successfully salvaged in eight patients with radial operations, 14 patients died of the disease, and the 5-year DSS rate was 90%. The two groups had similar RC (*p* = 0.731) and DSS (*p* = 0.583) rates (Figures 1, 2, both *p* > 0.05).

**Table 2:** Neck recurrence pattern in the two groups.

**Metastatic level**	**Group A (*n* = 9)**	**Group B (*n* = 20)**
Ipsilateral		
I	4 (44.4%)	8 (40.0%)
II	2 (22.2%)	6 (20.0%)
III	2 (22.2%)	0
IV	0	3 (15.0%)
V	0	1 (5.0%)
Contralateral		
I	1 (11.1%)	1 (5.0%)
II	2 (22.2%)	2 (10.0%)
III	0	0
IV	0	1 (5.0%)
V	0	1 (5.0%)

## Discussion

The most significant finding in the current study was that, compared to elective neck dissection, PET-CT–guided neck dissection obtained similar disease control of cT1-2N0 tongue SCC; moreover, there was a higher possibility of successful salvage with PET-CT–guided neck dissection than with elective neck dissection. Our study may provide more suggestions for neck management during early stage tongue SCC treatment, and it provides the first evidence for the high reliability of PET-CT–guided neck dissection.

Neck lymph node status is the most important prognostic factor; usually, the survival rate decreases by half if lymph node metastasis is present (19–21). It is important that surgeons correctly address the neck. However, owing to the varying occult metastasis rates, there is still substantial controversy regarding the best neck management. Canis et al. (4) studied early stage SCC of the upper aerodigestive tract, and the 5-year recurrence-free survival rate was 95% in the neck dissection group and 96% in the wait-and-see group; the authors concluded that a wait-and-see approach was justified in patients with early stage disease. Deganello et al. (22) described that the occult neck disease rate was only 12.5% for laryngeal SCC, and the findings indicated the need to refine the treatment strategy, restricting elective neck dissection only to advanced stage disease. However, tongue SCC was significantly different from SCC in other sites, and the conclusions based on the overall sample of head and neck SCC or laryngeal SCC might not be suitable for tongue SCC. Moratin et al. (23) depicted that regional metastases occurred in 29 of the 97 patients with early stage tongue SCC, and although rare, bilateral cervical metastases could be detected in 2.1% of T1 and 11.8% of T2 disease; therefore, the authors suggested that neck dissection was required in all tongue SCC patients irrespective of tumor stage. Recent evidence showed that, after matched analysis in 1,234 patients, the 5-year overall survival rates were 87.1% in the neck dissection group and 76.2% in the wait-and-see group (*p* = 0.0051), and the DSS rates were 89.2% and 82.2% (*p* = 0.0335), respectively. This finding suggests that routine neck dissection was beneficial for cT1-2N0 tongue SCC (24). A similar viewpoint was also demonstrated by Gad et al. (25), Orabona et al. (26), and D'Cruz et al. (1). However, at least two aspects must be considered seriously when comprehending the conclusions: First, the occult metastasis rate was variable in early stage tongue SCC, and most of the patients would have been over-treated if routine neck dissection was performed; second, the so-called survival difference between elective neck dissection and the wait-and-see policy would have been mainly explained by the undetected real metastatic lymph nodes at the first visit in the wait-and-see group. This raised the point that, if we could identify patients who had real metastatic lymph nodes preoperatively, we might avoid unnecessary neck dissection and achieve similar survival compared to routine neck dissection.

A number of authors have aimed to assess the predictors of occult lymph node metastasis in cT1-2N0 tongue SCC. Jiang et al. (6) introduced a nomogram including the site of the primary lesion, depth of invasion, size of the tumor, and histopathological grade with high consistency for predicting the probability of occult cervical metastasis before surgery. Tam et al. (7) demonstrated that a DOI of 7.25 mm was most predictive for occult nodal disease. Other predictors included PNI and LVI (1). However, the data were always unavailable preoperatively, which greatly limited the clinical application.

PET-CT has been widely used to detect occult lymph node disease in the clinic. Slightly conflicting results have been reported by previous researchers. Stoeckli et al. (27) assessed the value of PET-CT for the staging of cN0 necks in oral and oropharyngeal SCC using sentinel lymph node biopsy and elective neck dissection as the “gold standard” for comparison, and sentinel lymph node biopsy was accurately feasible in all 12 patients and correctly diagnosed five cases of occult metastasis; however, PET-CT only suggested that two patients had occult metastasis, of whom one turned out to be a false positive case. A similar finding was also reported by Krabbe et al. (28), but both studies did not specifically focus on tongue SCC, which is significantly different from SCC in other sites, and HPV infection might also affect the accuracy of PET-CT. Increasing evidence supports the high reliability of PET-CT for identifying cervical nodal disease. Zhang et al. (9) described that the overall sensitivity and specificity of PET-CT for predicting lymph node metastasis in cT1-2 oral SCC was 21.4 and 98.4%, respectively, with a negative predictive value of 99.1%. Myers et al. (10) presented an estimated overall sensitivity of PET-CT for the N0 neck of 78% with a specificity of 100%. Similar results were also reported by Zheng et al. (11), Lee et al. (12), and Wong et al. (13). A recent important study by Lowe et al. (29) described that PET-CT had a highly negative predictive value for the N0 neck in T2 to T4 SCC of the head and neck and that PET/CT might assist clinicians with the decision of the best therapy for the clinically N0 neck in SCC of the head and neck. Our results supported most of those of other researchers and verified the high reliability of PET-CT for predicting occult metastasis in cT1-2 disease, which could result in similar oncologic outcomes as routine neck dissection. The two groups had comparable important variables except the clinical tumor stage; patients undergoing a PET-CT–guided neck dissection tended to have a smaller tumor, it indicated that we selectively suggested a PET-CT for a small tumor to avoid unnecessary neck dissection and obtain a better functional result previously. Additionally, the tending difference of clinical tumor stage might have led to survival difference between the two groups, but in fact, it was not. It brought some puzzle in comprehending our finding; more high quality research is needed to clarify these questions.

Another heated debated tool of detecting occult metastasis was sentinel lymph node biopsy (SLNB). Moya-Plana et al. (30) compared the reliability between SLNB and END in 229 cT1-2N0 oral SCC patients; the authors reported negative predictive value of SLNB was as high as 92.7%, and the two groups had similar recurrence-free survival and overall survival. Riese et al. (31) described in 36 patients with early oral and oropharynx SCC, all the 12 patients with pathologic metastasis were detected in SLNB, and it showed a sensitivity of 94.4% and a specificity of 100%. All these findings as well as recent research (32–34) confirmed SLNB was a precise diagnostic procedure in oral SCC and superior to PET-CT in melanoma and anal cancer for assessing nodal status. However, comparison between SLNB and PET-CT in oral SCC was never reported, and also application of SLNB in oral cancer was not widely accepted in China. More high-quality studies are needed to clarify these questions.

Salvage operations are important for improving survival, but the rate of successful salvage operations is usually <50% in tongue SCC patients (1–3). A similar finding was also noted in our END group. However, in group A, our salvage operation rate was as high as 66.7%, and it tended to be higher than that in group B. This finding was interesting: The high salvage operation rate might have resulted in improved DSS, but owing to our limited sample size, we could not draw any persuasive conclusions.

It was interesting to note the occult metastasis rate of Group B was just 17.8%; it was a little lower than those of similar studies (35, 36). The difference might be partially explained by different pathologic DOI and races. On the other hand, micrometastasis disease was also partially responsible during standard haematoxylin and eosin; van den Brekel et al. (37) previously presented that 25% of patients with positive lymph nodes had micrometastasis and were at high risk for undetected disease. Similar findings were also reported by Barrera et al. (38) and Rhee et al. (39).

There are some limitations that must be acknowledged. First, this was a retrospective study, it lacked of randomization, and the inherent bias could decrease the statistical power. In our future work, we will perform a prospective study with additional data regarding patient characteristics, especially other preoperative investigations, which could increase the predictive value of negative or positive PET-CT results. Second, our sample size was small, and a study with a larger sample size is needed. Third, some confounding factors might not have been taken into consideration, such as oral hygiene.

In summary, neck dissection could be safely avoided when the PET-CT scan reveals no neck lymph node involvement.

## Data Availability Statement

The raw data supporting the conclusions of this article will be made available by the authors, without undue reservation, upon a reasonable request.

## Ethics Statement

The Zhengzhou University Institutional Research Committee approved our study, and all patients signed informed consent agreements for medical research before initial treatment.

## Author Contributions

All the authors make the contribution in data collection, data analysis, and manuscript writing.

## Conflict of Interest

The authors declare that the research was conducted in the absence of any commercial or financial relationships that could be construed as a potential conflict of interest.

## References

[1] D'Cruz AK Vaish R Kapre N Dandekar M Gupta S Hawaldar R Agarwal JP. Elective versus therapeutic neck dissection in node-negative oral cancer. N Engl J Med. (2015) 373:521–9. 10.1056/NEJMoa1506007 26027881

[2] Abu-Ghanem S Yehuda M Carmel NN Leshno M Abergel A Gutfeld O. Elective neck dissection vs observation in early-stage squamous cell carcinoma of the oral tongue with no clinically apparent lymph node metastasis in the neck: a systematic review and meta-analysis. JAMA Otolaryngol Head Neck Surg. (2016) 142:857–65. 10.1001/jamaoto.2016.1281 27442962

[3] Liu JY Chen CF Bai CH. Elective neck dissection versus observation in early-stage (cT1/T2N0) Oral squamous cell carcinoma. Laryngoscope Investig Otolaryngol. (2019) 4:554–61. 10.1002/lio2.301 31637301 PMC6793606

[4] Canis M Plüquett S Ihler F Matthias C Kron M Steiner W. Impact of elective neck dissection vs observation on regional recurrence and survival in cN0-staged patients with squamous cell carcinomas of the upper aerodigestive tract. Arch Otolaryngol Head Neck Surg. (2012) 138:650–5. 10.1001/archoto.2012.1026 22801889

[5] Borgemeester MC van den Brekel MW van Tinteren H Smeele LE Pameijer FA van Velthuysen ML. Ultrasound-guided aspiration cytology for the assessment of the clinically N0 neck: factors influencing its accuracy. Head Neck. (2008) 30:1505–13. 10.1002/hed.20903 18704967

[6] Jiang Q Tang A Long S Qi Q Song C Xin Y. Development and validation of a nomogram to predict the risk of occult cervical lymph node metastases in cN0 squamous cell carcinoma of the tongue. Br J Oral Maxillofac Surg. (2019) 57:1092–7. 10.1016/j.bjoms.2019.09.024 31677799

[7] Tam S Amit M Zafereo M Bell D Weber RS Depth of invasion as a predictor of nodal disease and survival in patients with oral tongue squamous cell carcinoma. Head Neck. (2019) 41:177–84. 10.1002/hed.25506 30537401

[8] Faisal M Abu Bakar M Sarwar A Adeel M Batool F Malik KI. Depth of invasion (DOI) as a predictor of cervical nodal metastasis and local recurrence in early stage squamous cell carcinoma of oral tongue (ESSCOT). PLoS ONE. (2018) 13:e0202632. 10.1371/journal.pone.0202632 30133515 PMC6105019

[9] Zhang H Seikaly H Biron VL Jeffery CC. Utility of PET-CT in detecting nodal metastasis in cN0 early stage oral cavity squamous cell carcinoma. Oral Oncol. (2018) 80:89–92. 10.1016/j.oraloncology.2018.04.003 29706193

[10] Myers LL Wax MK Nabi H Simpson GT Lamonica D. Positron emission tomography in the evaluation of the N0 neck. Laryngoscope. (1998) 108:232–6. 10.1097/00005537-199802000-00014 9473074

[11] Zheng D Niu L Liu W Zheng C Yan R Gong L. Relationship between the maximum standardized uptake value of fluoro-2-deoxyglucose-positron emission tomography/computed tomography and clinicopathological characteristics in tongue squamous cell carcinoma. J Cancer Res Ther. (2019) 15:842–8. 10.4103/jcrt.JCRT_855_18 31436241

[12] Lee SJ Choi JY Lee HJ Baek CH Son YI Hyun SH. Prognostic value of volume-based (18)F-fluorodeoxyglucose PET/CT parameters in patients with clinically node-negative oral tongue squamous cell carcinoma. Korean J Radiol. (2012) 13:752–9. 10.3348/kjr.2012.13.6.752 23118574 PMC3484296

[13] Wong WL Sonoda LI Gharpurhy A Gollub F Wellsted D Goodchild K. 18F-fluorodeoxyglucose positron emission tomography/computed tomography in the assessment of occult primary head and neck cancers–an audit and review of published studies. Clin Oncol. (2012) 24:190–5. 10.1016/j.clon.2011.11.001 22183080

[14] Zhao G Sun J Zhang Y Ba K. Significance of PET-CT for detecting occult lymph node metastasis and affecting prognosis in early-stage tongue squamous cell carcinoma. Front Oncol. (2020) 10:368. 10.3389/fonc.2020.00386 32328452 PMC7160696

[15] Ouyang PY Su Z Mao YP Liang XX Liu Q Xie FY. Prognostic impact of family history in southern Chinese patients with undifferentiated nasopharyngeal carcinoma. Br J Cancer. (2013) 109:788–94. 10.1038/bjc.2013.343 23807164 PMC3738126

[16] Fang QG Shi S Liu FY Sun CF. Squamous cell carcinoma of the oral cavity in ever smokers: a matched-pair analysis of survival. J Craniofac Surg. (2014) 25:934–7. 10.1097/SCS.0000000000000710 24820715

[17] Skulsky SL O'Sullivan B McArdle O Leader M Roche M Conlon PJ. Review of high-risk features of cutaneous squamous cell carcinoma and discrepancies between the American Joint Committee on cancer and NCCN clinical practice guidelines in oncology. Head Neck. (2017) 39:578–94. 10.1002/hed.24580 27882625

[18] Lydiatt WM Patel SG O'Sullivan B Brandwein MS Ridge JA Migliacci JC. Head and Neck cancers-major changes in the American Joint Committee on cancer eighth edition cancer staging manual. Cancer J Clin. (2017) 67:122–37. 10.3322/caac.21389 28128848

[19] Fang Q Li P Qi J Luo R Chen D Zhang X Value of lingual lymph node metastasis in patients with squamous cell carcinoma of the tongue. Laryngoscope. (2019) 129:2527–30. 10.1002/lary.27927 30861130

[20] Dai L Fang Q Li P Liu F Zhang X. Oncologic outcomes of patients with sarcomatoid carcinoma of the hypopharynx. Front Oncol. (2019) 9:950. 10.3389/fonc.2019.00950 31608238 PMC6769101

[21] Du W Fang Q Wu Y Wu J Zhang X. Oncologic outcome of marginal mandibulectomy in squamous cell carcinoma of the lower gingiva. BMC Cancer. (2019) 19:775. 10.1186/s12885-019-5999-0 31387576 PMC6683491

[22] Deganello A Gitti G Meccariello G Parrinello G Mannelli G Gallo O. Effectiveness and pitfalls of elective neck dissection in N0 laryngeal cancer. Acta Otorhinolaryngol Ital. (2011) 31:216–221. 22058599 PMC3203726

[23] Moratin J Metzger K Engel M Hoffmann J Freudlsperger C Freier K. The occurrence of cervical metastases in squamous cell carcinoma of the tongue: Is there a rationale for bilateral neck dissection in early-stage tumors? J Craniomaxillofac Surg. (2019) 47:1134–8. 10.1016/j.jcms.2019.03.003 30914228

[24] Otsuru M Ota Y Yanamoto S Okura M Umeda M Kirita T. A multicenter retrospective study of elective neck dissection for T1-2N0M0 tongue squamous cell carcinoma: analysis using propensity score-matching. Ann Surg Oncol. (2019) 26:555–63. 10.1245/s10434-018-07089-7 30515671 PMC6341049

[25] Gad ZS El-Malt OA El-Sakkary MAT Abdal Aziz MM. Elective neck dissection for management of early- stage oral tongue cancer. Asian Pac J Cancer Prev. (2018) 19:1797–803. 10.22034/APJCP.2018.19.7.1797 30049190 PMC6165647

[26] Orabona GD Bonavolontà P Maglitto F Friscia M Iaconetta G Califano L. Neck dissection versus “watchful-waiting” in early squamous cell carcinoma of the tongue our experience on 127 cases. Surg Oncol. (2016) 25:401–4. 10.1016/j.suronc.2016.09.005 27916172

[27] Stoeckli SJ Steinert H Pfaltz M Schmid S. Is there a role for positron emission tomography with 18F-fluorodeoxyglucose in the initial staging of nodal negative oral and oropharyngeal squamous cell carcinoma. Head Neck. (2002) 24:345–9. 10.1002/hed.10057 11933176

[28] Krabbe CA Balink H Roodenburg JL Dol J de Visscher JG. Performance of 18F-FDG PET/contrast-enhanced CT in the staging of squamous cell carcinoma of the oral cavity and oropharynx. Int J Oral Maxillofac Surg. (2011) 40:1263–70. 10.1016/j.ijom.2011.06.023 21824748

[29] Lowe VJ Duan F Subramaniam RM Sicks JD Romanoff J Bartel T. Multicenter trial of [18F]fluorodeoxyglucose positron emission tomography/computed tomography staging of head and neck cancer and negative predictive value and surgical impact in the N0 Neck: results from ACRIN 6685. J Clin Oncol. (2019) 37:1704–12. 10.1200/JCO.18.01182 30768363 PMC6638599

[30] Moya-Plana A Aupérin A Guerlain J Gorphe P Casiraghi O Mamelle G. Sentinel node biopsy in early oral squamous cell carcinomas: Long-term follow-up and nodal failure analysis. Oral Oncol. (2018) 82:187–94. 10.1016/j.oraloncology.2018.05.021 29909896

[31] Riese CGU Karstadt JA Schramm A Güleryüz S Dressel G Lorenz KJ. Validity of sentinel node biopsy in early oral and oropharyngeal carcinoma. J Craniomaxillofac Surg. (2018) 46:1748–52. 10.1016/j.jcms.2018.07.021 30145047

[32] Schilling C Shaw R Schache A McMahon J Chegini S Kerawala C. Sentinel lymph node biopsy for oral squamous cell carcinoma. Where are we now? Br J Oral Maxillofac Surg. (2017) 55:757–62. 10.1016/j.bjoms.2017.07.007 28864148

[33] El-Maraghi RH Kielar AZ. PET vs sentinel lymph node biopsy for staging melanoma: a patient intervention, comparison, outcome analysis. J Am Coll Radiol. (2008) 5:924–31. 10.1016/j.jacr.2008.02.022 18657789

[34] De Nardi P Guarneri G Canevari C Tamburini A Slim N Passoni P. Prognostic value of fluorodeoxyglucose positron emission tomography/computed tomography and inguinal sentinel lymph node biopsy in patients with anal cancer. Colorectal Dis. (2019) 21:1017–24. 10.1111/codi.14691 31077550

[35] Ahmed SQ Junaid M Awan S Kazi M Khan HU Halim S. Frequency of cervical nodal metastasis in early-stage squamous cell carcinoma of the tongue. Int Arch Otorhinolaryngol. (2018) 22:136–40. 10.1055/s-0037-1603626 29619101 PMC5882373

[36] Abbate V Dell'Aversana Orabona G Salzano G Bonavolontà P Maglitto F Romano A. Pre-treatment neutrophil-to-lymphocyte ratio as a predictor for occult cervical metastasis in early stage (T1-T2 cN0) squamous cell carcinoma of the oral tongue. Surg Oncol. (2018) 27:503–7. 10.1016/j.suronc.2018.06.002 30217309

[37] van den Brekel MW van der Waal I Meijer CJ Freeman JL Castelijns JA Snow GB. The incidence of micrometastases in neck dissection specimens obtained from elective neck dissections. Laryngoscope. (1996) 106:987–91. 10.1097/00005537-199608000-00014 8699914

[38] Barrera JE Miller ME Said S Jafek BW Campana JP Shroyer KR. Detection of occult cervical micrometastases in patients with head and neck squamous cell cancer. Laryngoscope. (2003) 113:892–6. 10.1097/00005537-200305000-00022 12792329

[39] Rhee D Wenig BM Smith RV. The significance of immunohistochemically demonstrated nodal micrometastases in patients with squamous cell carcinoma of the head and neck. Laryngoscope. (2002) 112:1970–4. 10.1097/00005537-200211000-00011 12439164

